# 

**Published:** 1877-07-20

**Authors:** 


☞All communications relating to the business of THE RECORD, for the year 1877, must be addressed to

DR. R. C. WORD,
Business Manger Southern Med. Rec.,
Atlanta, Ga.

☞Brief and practical communications are solicited on all subjects pertaining to medicine. also reports of cases in practice.

☞Send money by check, postal order or registered letter.

☞Write your name, post-o ffice, county and State plainly.

### COLLABORATORS AND CONTRIBUTORS.

| | | | |
|---|---|---|---|
| L. B. Anderson, M.D., Va. | E. T. Easley. A.M., M.D., Ark | Z. B. Herndon, M.D., Va. | C H Lathrop, M.D., Iowa |
| Swan M. Burnett, M.D., D. C | A. H. Goelet, M D., N. Y. | Lindsay Johnson, M.D., Ga. | A A Lyon, M.D., La. |
| J. M. Bristow M.D., Texas | E C. Gehrung, M.D., Mo. | A. R. Kilpatrick, M.D., Tex. | M Lewis, M.D., Tenn |
| J. W. Booth, M.D., N C. | G. D. Hodge, M.D., Ark. | R. Kells, M.D., Miss. | G M Rivers, M.D., S C |
| W. F. Barr, M.D., Va. | S. M. Hogan, M.D., Ala. | W. L. Lipscomb, M.D., Miss. | A Sayne, M.D., Va. |
| B. W. Corn, M.D., Texas | A. McLane Hamilton, M.D.,NY | J. M. Lewis, M.D., Miss. | A. M. Whitehead, M.D., O |
| John Castor, M.D., Iowa. | C. C. Vanderbeck, M.D., Pa. | G. A. Dyer, M.D., Ind. | |

